# Developing a framework for investigating citizen science through a combination of web analytics and social science methods—The CS Track perspective

**DOI:** 10.3389/frma.2022.988544

**Published:** 2022-10-05

**Authors:** Reuma De-Groot, Yaela N. Golumbic, Fernando Martínez Martínez, H. Ulrich Hoppe, Sally Reynolds

**Affiliations:** ^1^MOFET Institute - Research Center, Tel-Aviv, Israel; ^2^The Steinhardt Museum of Natural History, Tel Aviv University, Tel Aviv, Israel; ^3^Universidad Rey Juan Carlos (URJC), Escuela Técnica Superior de Ingeniería Informática, Madrid, Spain; ^4^RIAS - Rhein-Ruhr-Institut für angewandte Systeminnovation e.V., Duisburg, Germany; ^5^ATiT, Leuven, Belgium

**Keywords:** web-based analytics, social science analysis, citizen science, social networks analysis, content analysis

## Abstract

Over the past decade, Citizen Science (CS) has shown great potential to transform the power of the crowd into knowledge of societal value. Many projects and initiatives have produced high quality scientific results by mobilizing peoples' interest in science to volunteer for the public good. Few studies have attempted to map citizen science as a field, and assess its impact on science, society and ways to sustain its future practice. To better understand CS activities and characteristics, CS Track employs an analytics and analysis framework for monitoring the citizen science landscape. Within this framework, CS Track collates and processes information from project websites, platforms and social media and generates insights on key issues of concern to the CS community, such as participation patterns or impact on science learning. In this paper, we present the operationalization of the CS Track framework and its three-level analysis approach (micro-meso-macro) for applying analytics techniques to external data sources. We present three case studies investigating the CS landscape using these analytical levels and discuss the strengths and limitations of combining web-analytics with quantitative and qualitative research methods. This framework aims to complement existing methods for evaluating CS, address gaps in current observations of the citizen science landscape and integrate findings from multiple studies and methodologies. Through this work, CS Track intends to contribute to the creation of a measurement and evaluation scheme for CS and improve our understanding about the potential of analytics for the evaluation of CS.

## Introduction

Citizen Science (CS) is a growing phenomenon within scientific research, in which lay or non-scientists volunteer in scientific research activities. Well-known CS activities include butterfly counts, birdwatching, and monitoring air and water quality. Such projects have demonstrated the “power of the crowd” in delivering scientific, policy and social impact (Shirk et al., 2012; Turrini et al., 2018). The vast potential of CS has been demonstrated extensively in large scale projects which are mediated through online communities or apps that can accommodate many volunteers. Projects such as “Galaxy Zoo” which asks participants to visually classify pictures of galaxies, are quite successful regarding their scientific outcome (Golumbic et al., 2019). Apart from a high number of publications, the results are valuable for further research, for example the exploration of “Hanny's Voorwerp” in Galaxy Zoo (Lintott et al., 2009).

There is growing interest in the advancement of collaborative and co-creative projects which involve more responsibility on the part of the citizen scientists who are involved. In fact, a recent study conducted across 125 European-based projects found the roles citizen scientists undertook in the projects were predominantly those of collaborators (Moczek et al., 2021). Such approaches are increasingly chosen when attention is required from both local actors and communities in order to solve place-based problems and deliver community outcomes (Gunnell et al., 2021; Manzoni et al., 2021).

The growth of CS is apparent by the increasing number of projects on CS platforms such as Zooniverse, eu-citizen.science^1^ or scistarter^2^. Scistarter alone lists over 1,600 and the numbers are growing. CS growth is also reflected by the number of academic publications which have risen exponentially over the last two decades (Pelacho et al., 2021). Such publications include scientific findings derived from CS data, in addition to research on project design, infrastructure, benefits for participants, and more (Kullenberg and Kasperowski, 2016).

While several studies have attempted to map citizen science (e.g., Roy et al., 2012; Kullenberg and Kasperowski, 2016; Hecker et al., 2018), these are few and far between. Furthermore, despite the vast work that is currently taking place within CS projects and activities, there is still a lack of knowledge about the impact of these projects and activities on society and how to integrate CS into new policies. More information is needed about citizen engagement, appropriate research methodologies, and the contribution of CS to policy-making.

## The CS track project

The CS Track^3^ project aims to broaden our knowledge about CS from an “observer” perspective by combining web-analytics with quantitative and qualitative methods from social science practices. CS Track involves nine partners from seven countries with backgrounds in social and educational studies, computer science and data analytics, as well as research into CS. Such an approach strengthens the findings, provides more comprehensive data, increased validity, and ultimately enhances our understanding of the state of CS. Methods utilized by CS Track comprise literature reviews, content analysis (including web and social media content), exploring discourse related to CS in social networks and carrying out surveys and interviews with key stakeholders in the field.

CS Track has created a database of more than 4,500 CS projects. Although the database essentially comprises information on individual projects, the harvesting of project information used 56 global CS platforms as starting points, which enabled a partially automated approach using web crawling techniques. This database serves as a resource for our explorations, using descriptors and analytic methods to build a coherent understanding of CS based on big data exploration. The database structure is deliberately flexible and can be adjusted to incorporate emerging analysis results. CS Track has also conducted an online survey collecting subjective perceptions of participation and engagement of CS stakeholders with over 1,000 respondents. In some cases, CS Track utilizes interviews and content analysis methods. CS Track also shares its results with the wider CS community through an eMagazine, providing summaries of main findings and other outputs.

### Levels of analytics and analysis in CS track

It is a specificity of the CS Track project that it gains insight in CS practices by applying computational analytics techniques to existing websites and social media channels that contain manifestations of CS activities. Relevant computational techniques include data and text mining, semantic analyses (esp. “Explicit Semantic Analysis” or ESA, cf. Gabrilovich and Markovitch, 2007) as well as Social Network Analysis or SNA (Wasserman and Faust, 1994). The sources of primary information to be processed by analytics are human-created content in the form of natural language and formal texts. Some of this information, such as short descriptions of CS projects, is directly available in the CS Track database. Additional information can be gained by harvesting from CS platforms and project websites. Here, again, the database serves as an entry point providing indexical information such as names, acronyms, and web links. Techniques of Named Entity Recognition (NER) allow for extracting information on persons, institutions, geographical locations, etc. from given texts (Nadeau and Sekine, 2007). This information can be useful to locate-connections between projects and other institutions or support anonymization of personal information.

Given CS Track's observer perspective on a broad range of projects, there is no direct access to the internal processes and documents of individual CS projects, beyond their manifestations in project websites. Still, there is content information on websites that is even partly represented in the CS Track database (especially project descriptions). Interactions between project members can be retrieved from project-related forums and wiki pages, even in standardized form on platforms such as Zooniverse or SciStarter. This allows for building and analyzing network models from which influential actors or structural characteristics (e.g., hierarchical or reciprocal relationships) can be inferred. It also means that information about project content and activity can be captured. Depending on the level, the potential of applying analytic techniques to external data sources differs in the degree of automation that can be achieved. In this regard, we distinguish three different levels with specific characteristics:

(1) *Micro level applications*: Based on small samples using human judgement in the filtering and selection such as the analysis of communication and collaboration in project web forums for example, the analysis of the “Chimp & See” project on the Zooniverse platform which provided detailed insights in participation and role-taking between scientists and volunteers (Amarasinghe et al., 2021).
(2) *Meso level applications*: Here, we work with a predefined set of projects that allow for standardized, homogeneous data processing. The sampling (e.g., all projects from a particular platform) can be done through a simple filter applied to the CS Track database. For such a given sample, semantic analyses such as the identification of research areas or SDGs can be performed automatically.
(3) *Macro level applications*: Here, we harvest information from an open space that goes beyond the projects captured in the CS Track database. This allows for identifying special connections and trends related to the interplay between CS activities and a broader public. Such interactions take place in different social media of which the Twitter “blogosphere” is particularly suited as a source of analysis since it provides rich textual information with high potential for systematic search and retrieval (Mazumdar and Thakker, 2020). Network models can be built on the basis of different relations such as retweeting or following. Structural network analysis can be combined with content analysis of tweets.

## Case studies

The following case studies illustrate how these three levels of analytics are implemented in CS Track. Each includes a description of the methodologies used and insights that have emerged as a result. In-depth descriptions of each have been or are intended to be published elsewhere.

### Micro level: CS response to COVID-19 challenges

The COVID-19 pandemic has challenged scientists, researchers, and industries to rapidly divert their research to better understand the COVID-19 virus spread, biology and health implications in addition to identifying medical solutions and cures. One of the avenues utilized for this cause was CS.

In a micro level study, which involved a sample of CS projects, chosen by explicit criteria, we examined the power of CS to respond to emerging health challenges, through the example of the COVID-19 pandemic [see full report by Turbe et al. (2022)]. Twenty-Five CS projects were identified as conducting COVID-19 research, by searching the CS Track database and exploring COVID-19 dedicated projects lists produced by citizen science associations and research institutes globally (e.g., https://www.citizenscience.org/covid-19; https://www.wilsoncenter.org/blog-post/citizen-science-and-covid-19-power-distanced-crowd).

**Figure 1:**
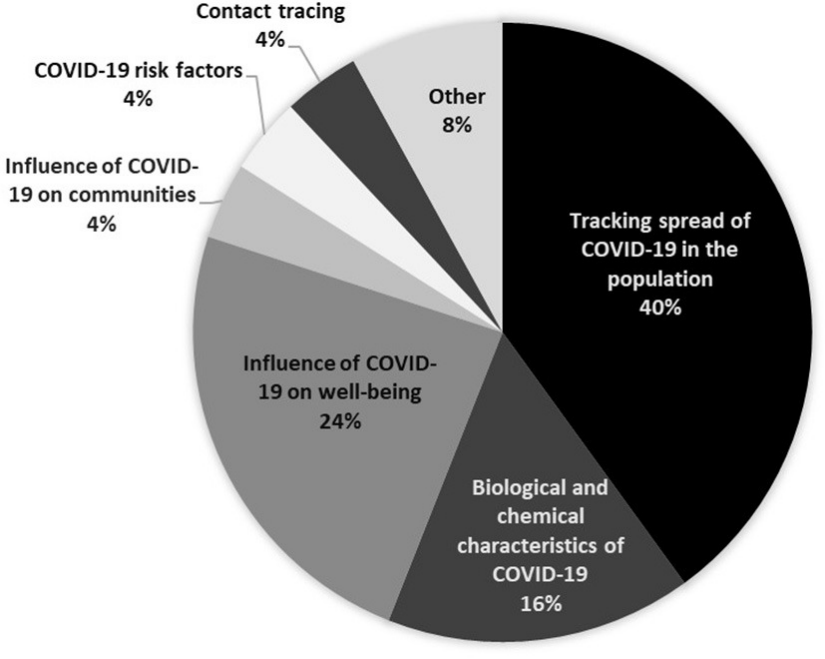
Primary aims of CS projects investigating COVID-19.

Content analysis of projects' websites revealed projects focused on three main domains, namely tracking the spread of the pandemic in the population, investigating the influence of COVID-19 on people's wellbeing, and investigating the COVID-19 virus biology (see Figure 1). Citizen scientists' tasks centered around responding to an online survey, self-tracking data from a wearable device and distributed computing. Overall projects were widely accessible, targeting a broad audience, and requiring no special skills. Most projects required at least a moderate degree of effort from participants, asking a few types of questions, and many required frequent contributions at regular intervals.

### Meso level: Identification of research areas for CS projects

Recent studies indicate that environmental sciences are a predominant research topic in the citizen science landscape (Follett and Strezov, 2015; Moczek et al., 2021). However, many of these analyses associate each project with only one main research area (Follett and Strezov, 2015; Lukyanenko et al., 2020), neglecting the multi-disciplinary nature of many projects. In a meso level study, which included a sample of all CS projects listed on the Zooniverse platform (*n* = 218), we have investigated the multi-disciplinarity nature of projects through an assessment of research areas within a subset of projects in the CS Track database.

To assign research areas to projects we relied on the ESA approach of semantic analysis (Gabrilovich and Markovitch, 2007). ESA combines statistical models with semantic background knowledge taken from Wikipedia pages. Every research area has a corresponding Wikipedia article in this model, which allows for the comparison of terms or documents regarding their semantic relation. By computing the similarities of project descriptions to research areas, it was possible to assign research areas to projects.

**Figure 2:**
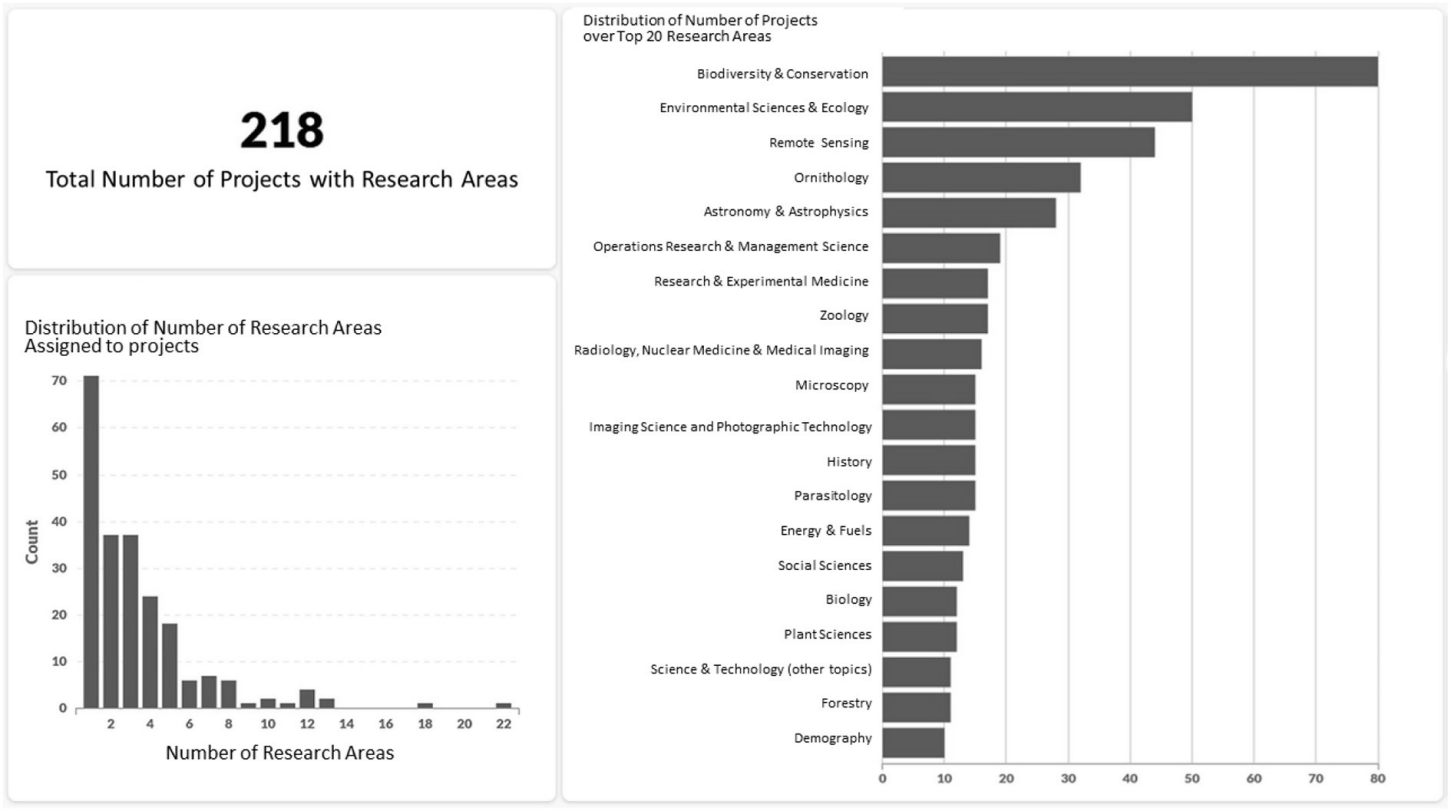
Dashboard visualizing the results of the research areas analysis for projects in the CS Track database. The selection of projects was limited to 218 Zooniverse projects.

Figure 2 shows a combined diagram of the results from this analysis based on 218 project descriptions taken from the Zooniverse platform. Notably, 147 of these projects (67.4%) have more than one associated research area. The average number of associated research areas is 3.34 and 11 projects have associations to 10 or more research areas. This shows that multi or inter-disciplinarity is a prevailing characteristic of CS projects.

### Macro level: CS on twitter

Following the macro level paradigm, Twitter data were used in a recent analysis of discussions related to climate change. Here, machine learning techniques for detecting sentiments were applied to tweets originating from within and outside the CS community. The analysis was based on the BERT approach (Devlin et al., 2018) using the multilingual uncased pretrained model, to cope with the presence of multiple languages in our dataset.

The data from within the CS community were extracted from a collection that had been created by Roldán-Álvarez et al. (2021), originally to detect connections that characterize the relation of CS activities to Sustainable Development Goals (SDGs). From this dataset, we extracted tweets about climate change by custom filtering using terms such as “sdg13,” “climate action,” “climate change,” or “climate justice.” This resulted in a dataset of 26,000 original tweets and 95,000 retweets. Using a corresponding search query, Tweets about climate change originating from outside the CS community were retrieved from the Twitter space. This resulted in 71,000 additional original tweets. For the sentiment analysis, a cropped version of the T4SA reference dataset (Vadicamo et al., 2017) with the same number of tweets labeled as negative, neutral, and positive was used as a training dataset.

The analysis of the original tweets (excluding retweets) revealed sentiments were mainly neutral within (92.9%) and outside (76.7%) the CS community. The ratio between positive and negative tweets was 1.8 (predominantly positive) within, and 0.89 (more or less balanced) outside. While these differences are less pronounced than we would expect from recent reports (Marlow et al., 2021; Moernaut et al., 2022), they still indicate that the climate change debate is less polarized within the CS community.

## Discussion and conclusions

Data analytics methodologies are widely used in research across scientific disciplines to assess and analyze current domain-related practices and scientific activities. For these purposes, big volumes of input data can be found in domain-specific archives but also in general web and social media sources. The contribution of CS projects and activities to generating and providing such data collections has been characterized by Poisson et al. (2020) for the area of geographic information systems and by Tang et al. (2017) for environmental big data. However, few efforts have been made to facilitate such approaches for analyzing the CS landscape as a whole, broadening our knowledge on the scope and state of CS to date. This is the perspective taken by the CS Track project.

A specific focus on scientific publications allows for using well-understood “scientometric” methods applied to available corpora of publication data. Kullenberg and Kasperowski (2016) have used a scientometric approach to identify the subjects or “focal points” of CS research activities, whereas, Pelacho et al. (2021) analyze co-publication networks to characterize and compare collaborations in the CS community in an international perspective. This enables the CS landscape to be addressed as a whole yet is limited to publication databases as data input and addresses only specific issues about CS. CS Track widens the scope by including data from different web and social media sources that capture external manifestations of CS activities.

The different levels of analysis introduced above are associated with typical data sources, including the CS Track database with project-related attributes and metadata, forum and wiki data available on CS platforms such as Zooniverse or SciStarter as well as the open blogosphere of Twitter. Among the data and metadata found in the database, are project descriptions or documentations that were usually written by authors themselves involved in these projects in leading roles. These meso-level descriptions account for expectations and goals “behind” these projects. Text-analytic methods, including sophisticated machine learning techniques, allow these descriptions to be associated with motivational factors or skill requirements. However, these analyses cannot reveal the individual motivation or learning gains of volunteers participating in these projects. A similar discrepancy occurs with the analytics of micro-level data from forums and webpages: These data allow for following general trajectories that may indicate “personal growth” (measured, e.g., in terms of increasing network centrality) or assess the distribution of tasks between professional scientists and volunteers in the discourse. However, they do not directly give us an account of subjective perceptions like feeling satisfied or rewarded by this work. To address the individual and subjective level, the analytics results have to be complemented with data from interviews or questionnaires from project participants or other contributors. The full analysis perspective in CS Track includes this integration (also called “triangulation”) as a current focus for the project.

Although micro level analyses like those based on participation data from forums and talk pages allow for identifying individual contributors, we would not use these for individual profiling in adherence to privacy-related ethical principles (cf. Cooper et al., 2021). Accordingly, our units of analysis are single projects, i.e., we characterize projects by certain participation patterns and make comparisons between projects on that basis. Results that rely on meso level content analyses such as the assignment of research areas (cf. Case 2) are also naturally related to projects or groups of projects. The scope of the Twitter-based macro level analysis is usually broader: Case 3 shows how the “climate of discourse” can be comparatively assessed between large sets of contributions within and outside the CS community as manifested in the Twitter blogosphere. A plausible explanation is that the context of the community discourse itself, which is more science-oriented within CS, induces a different tone or style.

An example for an analysis that integrates evidence from different sources comes from the COVID-19 study (Case 1). Following the analysis of web content, interviews have been conducted with key projects to complement the data and better understand projects' experiences researching COVID-19. This provided valuable information about project design, characteristics, and motivations in addition to reflections of project leaders on what actions have been successful and what can be improved in the future design and development of CS projects (Turbe et al., 2022). Ultimately, using mixed methods of analytics and social science provides methodological richness, allowing for the triangulation of data. That is a systematic comparison of data obtained from different sources and research perspectives which provides a coherent, validated, and holistic perspective of the CS landscape. This approach allows us to deepen our understanding about the main topics and concepts which are communicated over the web and characterize CS and the different ways it is perceived and approached by people who lead or take part in CS activities.

The CS Track framework aims to complement existing methods for evaluating CS, address gaps in current observations of the citizen science landscape and integrate findings from multiple studies and methodologies. The work done in this project so far and reported briefly above, should be seen as pioneering in its efforts to use mixed analytic and social science methods along with the triangulation of results from different sources, to achieve a broad picture of social phenomena related to the case of CS. Future work is expected to concentrate on refining the measurement and evaluation scheme for CS and summarize results of our analysis to provide further recommendations for best practices and policies for the CS community.

## Data availability statement

The datasets presented in this article are not readily available because restrictions apply to the original data under the GDPR regulation. The datasets will be made available on request.

## Author contributions

FM has contributed the macro-level case study. SR has been responsible for contributing to the article where it relates to the scientific communication aspect and for input in terms of text editing and language. YG was responsible for structuring of the paper, describing the study background, CS Track context and future directions. She has also contributed the micro-level case study together with RD-G. RD-G has been responsible for the organization of the writing and the design of the concept of the article. She was in charge of the work distribution amongst the partners. She is the first author and the corresponding author. She has contributed the micro-level case study together with YG. UH has been responsible for specifying and describing the technical basis of the analytics approach and has contributed the meso-level case study.

## Funding

The work in this paper has been carried out in the framework of the CS Track project (Expanding our knowledge on Citizen Science through analytics and analysis), which is funded by the European Commission under the Horizon 2020/SwafS program. Project reference number (Grant Agreement number): 872522.

## Conflict of interest

Author SR was employed by company ATiT. The remaining authors declare that the research was conducted in the absence of any commercial or financial relationships that could be construed as a potential conflict of interest.

## Publisher's note

All claims expressed in this article are solely those of the authors and do not necessarily represent those of their affiliated organizations, or those of the publisher, the editors, and the reviewers. Any product that may be evaluated in this article, or claim that may be made by its manufacturer, is not guaranteed or endorsed by the publisher.

## Footnotes

1 EU-Citizen Science. Available online at: https://eu-citizen.science/ (accessed 15 December, 2021).

2 Scistarter, Arizona State University. Available online at: https://scistarter.org/ (accessed 15 December, 2021).

3 CS Track European project. Available online at: http://cstrack.eu.
